# Prothrombotic State and Vascular Damage in Angiotensin II–Induced Hypertension: Influence of Waterpipe Smoke Exposure

**DOI:** 10.1155/omcl/2670738

**Published:** 2025-01-23

**Authors:** Sumaya Beegam, Suhail Al-Salam, Nur Elena Zaaba, Ozaz Elzaki, Abderrahim Nemmar

**Affiliations:** ^1^Department of Physiology, College of Medicine and Health Sciences, United Arab Emirates University, Al Ain P.O. Box 17666, UAE; ^2^Department of Pathology, College of Medicine and Health Sciences, United Arab Emirates University, Al Ain P.O. Box 17666, UAE; ^3^Zayed Center for Health Sciences, United Arab Emirates University, Al Ain P.O. Box 17666, UAE

**Keywords:** hypertension, thrombosis, vascular injury, waterpipe smoke

## Abstract

Hypertension is a risk factor for vascular injury and thrombotic complications, and smoking tobacco is a risk factor for the development and exacerbation of hypertension. The influence of waterpipe smoke (WPS) on coagulation and vascular injury in hypertension is not fully understood. Here, we evaluated the effects of WPS in mice made hypertensive (HT) by infusing angiotensin II (Ang II) for 42 days. On day 14 of the infusion of Ang II or vehicle (normotensive; NT), mice were exposed either to air or WPS for four consecutive weeks. Each session was 30 min/day for 5 days/week. The concentrations of tissue factor, von Willebrand factor, fibrinogen, and plasminogen activator inhibitor-1 were elevated in the HT + WPS group versus either HT + air or NT + WPS groups. Similarly, in the HT + WPS group, thrombogenicity was increased both in vivo and in vitro, compared with either HT + air or NT + WPS groups. In aortic tissue, adhesion molecules including P-selectin, E-selectin, intercellular adhesion molecule-1, and vascular adhesion molecule-1 were increased in the HT + WPS group versus the controls. Likewise, various proinflammatory cytokines and markers of oxidative stress augmented in the HT + WPS group compared with either HT + air or NT + WPS. DNA damage, cleaved caspase-3, and cytochrome C were increased in the HT + WPS group versus the controls. The immunohistochemical expression of nuclear factor erythroid 2-related factor 2 was increased in the HT + WPS group versus either HT + air or NT + WPS. Taken together, our findings show that WPS exposure intensified thrombogenicity and vascular damage in experimentally induced hypertension. Our data suggest that vascular toxicity of WPS may be exaggerated in hypertensive patients.

## 1. Introduction

Plaque rupture and superficial erosion usually lead to the formation of localized thrombi, often causing blockages. When this afflicts the coronary artery, it can result in downstream ischemia and myocardial infarction [1]. It is crucial to comprehend the processes triggering and potentiating this thrombus formation and the subsequent sequence of events [2]. Damage to the endothelial surface results in platelet adhesion, primarily due to platelet receptors binding to exposed collagen and von Willebrand factor (vWF) in the underlying matrix [3]. Additionally, platelets become activated through the coagulation pathway [1].

Hypertension impacts over 116 million adults in the United States and more than 1 billion adults globally [4]. This condition heightens the risk of cardiovascular diseases such as coronary heart disease, heart failure, and stroke and mortality [4]. It is well-established that hypertension is associated with irregularities in platelet activity, blood clotting, and fibrinolysis. The latter may potentially explain the elevated risk of thrombosis in individuals with this condition [5, 6]. The activated renin–angiotensin system and its main mediator, angiotensin II (Ang II), have been implicated in the prothrombotic state associated with hypertension [5, 6]. Additionally, administering Ang II to experimental animals mimics many of the platelet function irregularities and increased clotting observed in large arteries among patients with hypertension [7, 8].

Waterpipe smoking (WPS) has recently become a worldwide tobacco epidemic, particularly popular among young people [9, 10]. Various factors contribute to the increased popularity of WPS, compared with cigarette smoke, such as the use of flavoring that makes it more attractive, the misconception that it is less addictive and less harmful than conventional cigarette smoking, and that the water filtration system removes toxins [9, 10]. Substantial clinical and experimental evidence exists on the prothrombotic impact of WPS [11–13]. Nevertheless, as far as we know, no studies have reported the possible effect of WPS inhalation on thrombogenicity in experimental hypertension. Investigating such possible interaction is both important and clinically relevant, as it is well-established that the prevalence of active tobacco smoking is high among patients with hypertension or diabetes mellitus [14–16].

Therefore, the goal of this study was to evaluate, in a murine model of hypertension, the potential exacerbating impact of WPS inhalation on thrombogenicity and vascular inflammation, oxidative stress, DNA damage, and apoptosis and to elucidate the possible underlying mechanisms.

## 2. Material and Methods

### 2.1. Animals and WPS Exposure

This experimental work was approved by the Institutional Review Board of the UAE University, and was conducted in accordance with the ethics protocol #ERA_20195983.

BALB/c mice, both male and female and aged between 6 and 8 weeks, were procured from the animal house facility of the College of Medicine and Health Sciences, UAE University. They were accommodated in a standard animal house, following a 12-h light-dark cycle (lights on at 6 AM), and provided with additive-free pelleted food and water ad libitum. After an adaptation period of 1 week, the mice were randomly divided into four groups.

The study used an established animal model of Ang II–induced hypertension (HT) [8, 17]. To induce HT, mice were anesthetized with sodium pentobarbital (60 mg/kg, i.p.), and an Alzet osmotic pump Model 2006 (Mountain View, CA, USA) filled with angiotensin (ANG) II (0.75 mg/kg/day Ang II in 0.15 mol/L NaCl and 0.01 N acetic acid; Sigma–Aldrich Co., St. Louis, MO, USA; Catalog No. A9525) or vehicle was implanted in a subcutaneous pocket on the mouse's back and infused for 6 weeks. This dosage mirrored that of previous studies [8, 18].

At the end of the second-week postinfusion (day 14), the mice were gently restrained and connected to the WPS exposure tower. Both normotensive (NT) and HT mice were exposed for 4 weeks to either WPS or clean air via inhalation through their noses. This exposure was facilitated using a nose-only system attached to a waterpipe device (InExpose System, Scireq, Canada) [13, 19]. The animals were exposed to a commercially available apple-flavored tobacco (Al Fakher Tobacco Trading, Ajman, UAE), ignited with an instant light charcoal disk. Analogous to human exposure, the WPS initially passed through the water before being drawn into the WPS exposure tower. The exposure was precisely monitored using a computerized system (InExpose System, Scireq, Canada). A computer-regulated puff was generated every minute, consisting of 2 s of WPS exposure followed by 58 s of fresh air, and the total exposure duration was 30 min/day [13, 19]. This exposure period was determined based on previous human studies involving WPS [20].

### 2.2. Blood Pressure Assessment

The assessment of the systolic blood pressure (SBP) in mice was performed using a IN125 model computer-based-noninvasive tail-cuff manometry system purchased from ADInstrument (Colorado Springs, USA) [21]. To avert the stress induced by the technique, the animals were trained for five consecutive days prior to the day of the experiment. The measurement of the SBP was carried out at the start of the experiments, and after that on a weekly basis, for the whole length of the experimental protocol.

### 2.3. Blood Collection and Biochemical Analysis in Plasma

Following the measurement of SBP, mice were anesthetized with sodium pentobarbital (45 mg/kg) administered intraperitoneally. Approximately 800 µL of blood was then withdrawn in EDTA (4%) from the inferior vena cava and spun at 900 g for 15 min at 4°C. The resulting plasma samples were stored at −80°C until the analysis of tissue factor (using an ELISA kit from R&D Systems, Minneapolis, MN, USA; Catalog No. DY3178), vWF (using an ELISA kit from Cloud Clone, Katy, TX, USA; Catalog No. SEA833Mu), fibrinogen (kits obtained from Elabscience, Houston, TX, USA; Catalog No. E-EL-M0498), and plasminogen activator inhibitor-1 (using an ELISA kit from R&D Systems, Minneapolis, MN, USA; Catalog No. DY3828).

### 2.4. Experimental Pial Microvascular Thrombosis Model

The in vivo pial microvascular thrombosis was assessed in pial arterioles and venules at the completion of the exposure period to either WPS or air in NT and HT animals as reported before [13, 19].

### 2.5. Platelet Aggregation in Whole Blood In Vitro

The platelet aggregation assay was conducted using whole blood from a separate group of NT and mice exposed either to air or WPS following the previously described method [13, 19]. After anesthesia, ~800 µL of blood was collected from the *vena cava* and anticoagulated with citrate (3.2%). Subsequently, aliquots (100 µL) were added to the wells of a Merlin coagulometer (MC 1 VET, Merlin, Lemgo, Germany). The samples were then incubated with 0.1 µM adenosine 5′-diphosphate sodium salt (ADP; Sigma–Aldrich Co.; St. Louis, MO, USA; Catalog No. A2754) for 3 min at 37.2°C and stirred for an additional 3 min. After incubation, samples (25 µL) were taken and immediately fixed on ice in cellFix (225 mL) (Becton Dickinson, Franklin Lakes, NJ). Platelet aggregation, induced by ADP, led to a reduction in the count of individual platelets in blood samples analyzed using the ABX VET ABC hematology analyzer with a mouse-specific card (ABX Diagnostics, Montpellier, France). This assessment was performed on blood collected from both NT and HT animals exposed to either air or WPS. The results were compared with untreated (non-ADP-treated) whole blood from unexposed control mice [13, 19].

### 2.6. Prothrombin Time (PT) and Activated Partial Thromboplastin Time (aPTT) in Plasma In Vitro

The PT and aPTT were measured in plasma collected from NT and HT mice exposed either to air or WPS using TEClot PT-S (Catalog No. A0230-040) and TEClot aPTT-S (Catalog No. A0300-025) kits (TECO GmbH, NB, Germany), following the manufacturer's guidelines. Briefly, platelet-poor plasma was preincubated at 37°C for 3 min and then combined with PT and aPTT reagents. The measurements were performed using a Merlin coagulometer (MC 1 VET, Merlin, Lemgo, Germany) [19].

### 2.7. Quantification of Adhesion Molecules and Markers of Oxidative Stress, Inflammation, and Apoptosis in Aortic Homogenates

The aortic homogenates were prepared for the measurements of adhesion molecules and markers of oxidative stress, inflammation, and apoptosis, as described earlier [22]. The quantification of the concentrations of e- and P-selectins (Catalog Nos. DY575 and DY737), vascular cell adhesion molecule-1 (VCAM-1; Catalog No. DY643), and intercellular adhesion molecule-1 (ICAM-1; Catalog No. DY796) in aortic homogenates was carried out using Elisa kits procured from R&D systems (Minneapolis, MN, USA). Likewise, the ELISA kits used to measure the concentrations of tumor necrosis factor *α* (TNF*α*; Catalog No. DY410), interleukin (IL)-1*β* and IL-6 (Catalog No. DY401 and DY406) were purchased from the same company. 8-Isoprostane concentration and catalase activity (kits from Cayman Chemicals, Michigan, USA; Catalog No. 516351 and 707002) were measured according to the vendor's protocol. The quantification of nitric oxide (NO) was achieved with a colorimetric technique that measures more stable NO metabolites NO_2_^−^ and NO_3_^−^ [23]. Apoptotic markers including cleaved caspase-3 and cytochrome C were measured using commercially available kits obtained from R&D systems (Minneapolis, MN, USA; Catalog No. DYC835) and MyBiosource (San Diego, CA, USA; Catalog No. MBS2088690).

### 2.8. DNA Damage Assessment in Aortic Homogenates

DNA damage assessment through the COMET assay was conducted in distinct groups of mice right after their sacrifice. Aortae were collected and processed to evaluate DNA damage using the COMET technique, following established protocols [13]. DNA migration, including nucleus diameter and migrated DNA, was analyzed using the Axiovision 3.1 software (Carl Zeiss, Toronto, ON, Canada), as previously outlined [13].

### 2.9. Histology and Immunohistochemistry

The aortae, fixed in buffered formalin (10%), were removed, rinsed with ice-cold saline, dried with filter paper, and weighed. Each aorta was then placed in cassettes, dehydrated in escalating ethanol concentrations, cleared with xylene, and embedded in paraffin. Sections of 3 μm were cut from the paraffin blocks and stained with hematoxylin and eosin.

For immunohistochemistry, 5-μm aortic sections were prepared and mounted on aminopropyltriethoxysilane (APES) coated slides. After dewaxing with xylene and rehydrating with graded alcohol, slides were placed in a retrieval solution (pH = 9.0) and pretreatment procedures to unmask the antigens were performed in a water bath for 30 min at 95°C. Then, sections were treated with peroxidase block for 10 min. Sections were incubated with antinuclear factor erythroid 2–related factor 2 (Nrf2) (Rabbit Polyclonal, 1 : 100, Abcam, USA; Catalog No. AB76026), for 1 h at room temperature (RT). After conjugation with the primary antibody, sections were incubated with a secondary antibody (EnVision Detection System, DAKO, Agilent, USA) for 20 min at RT followed by the addition of DAB chromogen (EnVisionTM Detection System, DAKO, Agilent, USA) and counter staining done with hematoxylin. Appropriate positive controls were used. For negative control, the primary antibody was not added to sections. Positive and negative controls were used in every batch of slides that were stained (not shown in figures). The immunohistochemical staining of the aortas was scored according to the % of staining of each section of the aorta [24, 25].

### 2.10. Statistics

Statistical analyses were performed with GraphPad Prism Software version 7. Comparisons between groups were performed by one-way analysis of variance (ANOVA), followed by Holm–Sidak's multiple comparisons test. All the data in the figures and table were reported as mean ± SD. *p* values < 0.05 are considered significant.

## 3. Results

### 3.1. Assessment of SBP Following Exposure to WPS in NT and HT Mice

As shown in Table 1, exposure to WPS significantly increased SBP in NT mice compared to those exposed to air (*p*  < 0.0001). In HT mice, WPS exposure also caused a significant increase in SBP, which was higher compared to both the HT + air group (*p*  < 0.0001) and the NT + WPS group (*p*  < 0.0001). Furthermore, SBP was significantly higher in the HT + air group compared to the NT + air group (*p*  < 0.0001).

### 3.2. Tissue Factor, vWF, Fibrinogen, and PAI-1 Concentrations in the Plasma Following the Exposure to WPS in NT and HT Mice

**Figure 1:**
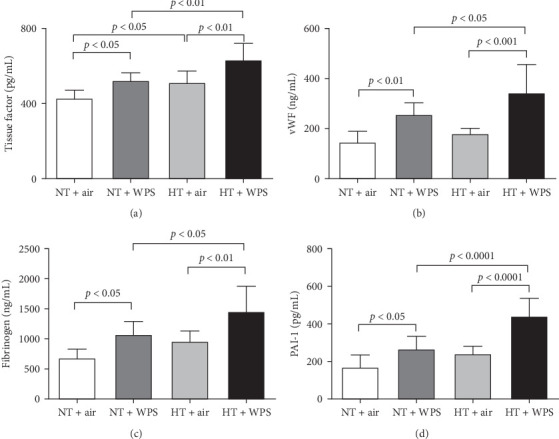
Tissue factor (A), von Willebrand factor (vWF; B), fibrinogen (C), and plasminogen activator inhibitor-1 (PAI-1; D) in plasma following exposure to either air or waterpipe smoke (WPS) in normotensive (NT) and hypertensive (HT) mice. Data are mean ± SD (*n* = 7−8 in each group). Statistical analysis by one-way ANOVA followed by Holm–Sidak's multiple comparisons test. ANOVA, analysis of variance.

As shown in Figure 1, compared to NT group exposed to air, the exposure to WPS in NT mice induced a significant increase in the concentrations of tissue factor, vWF, fibrinogen, and PAI-1 in the plasma indicating a procoagulant action of WPS (*p*  < 0.01 − *p*  < 0.05) (Figure 1). The exposure to WPS in HT mice induced a significant increase in the concentrations of tissue factor, vWF, fibrinogen, and PAI-1 in the plasma compared with HT + air (*p*  < 0.0001 − *p*  < 0.01) and NT + WPS (*p*  < 0.0001 − *p*  < 0.05) groups (Figure 1).

### 3.3. *In Vivo* Thrombotic Occlusion Time in Pial Arterioles and Venules Following the Exposure to WPS in NT and HT Mice

**Figure 2:**
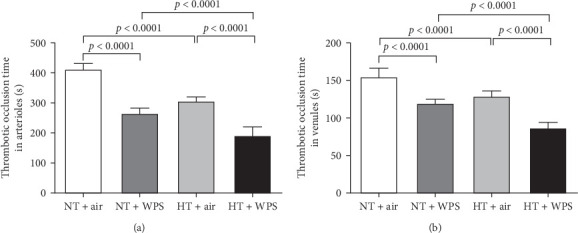
Thrombotic occlusion time in pial arterioles (A) and venules (B) following exposure to either air or waterpipe smoke (WPS) in normotensive (NT) and hypertensive (HT) mice. Data are mean ± SD (*n* = 8 in each group). Statistical analysis by one-way ANOVA followed by Holm–Sidak's multiple comparisons test. ANOVA, analysis of variance.

Figure 2 shows that compared with NT + air group, the exposure to WPS in NT mice caused a significant decrease of the thrombotic occlusion time in pial arterioles (*p*  < 0.0001) and venules (*p*  < 0.0001) indicating an increase in thrombogenicity. Likewise, the thrombotic occlusion time in HT + air was shortened compared with NT + air in both arterioles (*p*  < 0.0001) and venules (*p*  < 0.0001). HT mice exposed to WPS showed a significant reduction in the thrombotic occlusion time in both arterioles and venules when compared with either HT + air (*p*  < 0.0001) or NT + WPS (*p*  < 0.0001).

### 3.4. *In Vitro* Platelet Aggregation in Whole Blood Following the Exposure to WPS in NT and HT Mice

**Figure 3:**
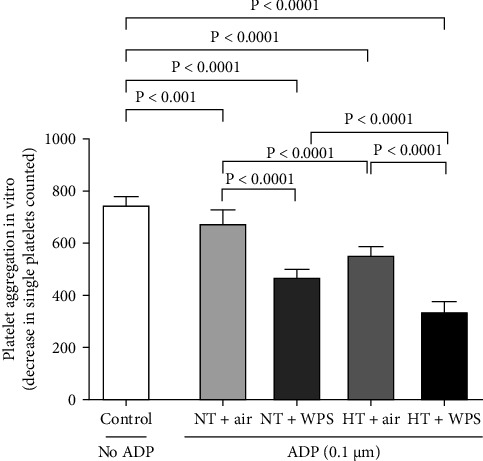
Platelet aggregation in whole blood in the presence or absence of adenosine diphosphate (ADP, 0.1 µM). The blood was collected from mice following exposure to either air or waterpipe smoke (WPS) in normotensive (NT) and hypertensive (HT) mice. Platelet aggregation was quantified by measuring the decrease in single platelets counted due to aggregation induced by ADP. Data are mean ± SD (*n* = 8 in each group). Statistical analysis by one-way ANOVA followed by Holm–Sidak's multiple comparisons test. ANOVA, analysis of variance.

Figure 3 illustrates that the incubation with ADP of whole blood collected from NT + WPS mice showed more platelet aggregation compared with NT + air (*p*  < 0.0001). Moreover, following the incubation with ADP, there was a more significant platelet aggregation in the whole blood of HT mice exposed to WPS compared with that collected from either HT + air (*p*  < 0.0001) or HT + WPS (*p*  < 0.0001).

### 3.5. *In Vitro* Assessment of PT and aPTT Following the Exposure to WPS in NT and HT Mice

**Figure 4:**
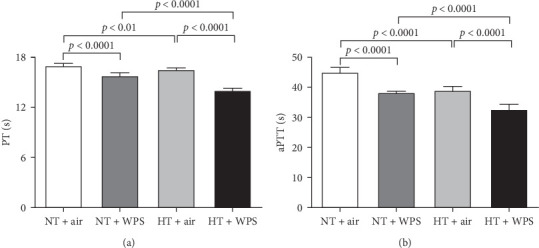
Prothrombin time (PT; A) and activated partial thromboplastin time (aPTT; B) following exposure to either air or waterpipe smoke (WPS) in normotensive (NT) and hypertensive (HT) mice. Data are mean ± SD (*n* = 8 in each group). Statistical analysis by one-way ANOVA followed by Holm–Sidak's multiple comparisons test. ANOVA, analysis of variance.

As illustrated in Figure 4, there was significant shortening in the PT and aPTT measured in NT + WPS group compared with that assessed in NT + air (*p*  < 0.0001). Additionally, PT and aPTT were further reduced in HT mice treated with WPS versus HT animals treated with air and NT mice treated with WPS (*p*  < 0.0001).

### 3.6. P-Selectin, E-Selectin, ICAM-1, and VCAM-1 Concentrations in the Aorta Following the Exposure to WPS in NT and HT Mice

**Figure 5:**
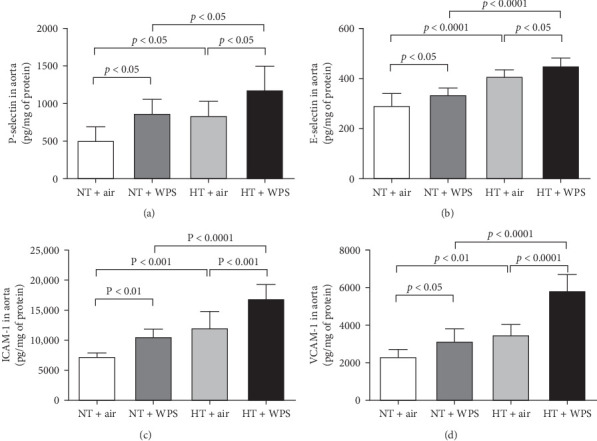
P-selectin (A), E-selectin (B), intercellular adhesion molecule-1 (ICAM-1; C), and vascular adhesion molecule-1 (VCAM-1; D) in aortic tissue following exposure to either air or waterpipe smoke (WPS) in normotensive (NT) and hypertensive (HT) mice. Data are mean ± SD (*n* = 7−8 in each group). Statistical analysis by one-way ANOVA followed by Holm–Sidak's multiple comparisons test. ANOVA, analysis of variance.

In comparison with NT + air, the concentrations of P-selectin, E-selectin, ICAM-1, and VCAM-1 were significantly increased in the aortic homogenates of NT + WPS (*p*  < 0.01 − *p*  < 0.05). Similarly, the concentrations of P-selectin, E-selectin, ICAM-1, and VCAM-1 were significantly increased in HT + air mice versus NT + air group (*p*  < 0.0001–*p*  < 0.05). Furthermore, P-selectin, E-selectin, ICAM-1, and VCAM-1 concentrations were significantly augmented in HT + WPS group versus HT + air (*p*  < 0.0001–*p*  < 0.05) and NT + WPS (*p*  < 0.0001–*p*  < 0.05) groups (Figure 5).

### 3.7. TNF*α*, IL-1*β*, and IL-6 Concentrations in the Aorta Following the Exposure to WPS in NT and HT Mice

**Figure 6:**
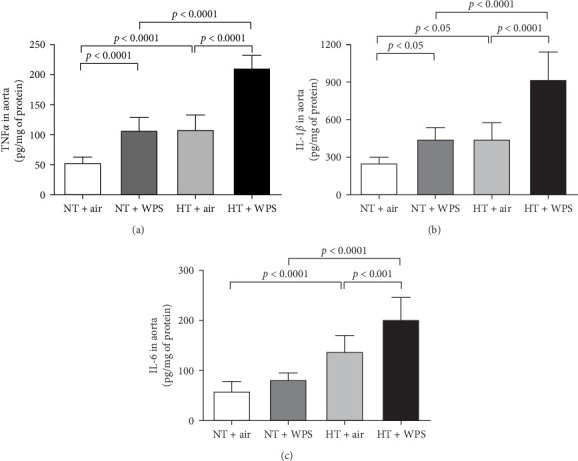
Tumor necrosis factor *α* (TNF*α*; A), interleukin (IL)-6 (B), and IL-1*β* (C) in aortic tissue following exposure to either air or waterpipe smoke (WPS) in normotensive (NT) and hypertensive (HT) mice. Data are mean ± SD (*n* = 8 in each group). Statistical analysis by one-way ANOVA followed by Holm–Sidak's multiple comparisons test. ANOVA, analysis of variance.

The concentrations of TNF*α* and IL-1*β* in the aortic tissue homogenates were significantly increased in NT + WPS versus NT + air (*p*  < 0.0001–*p*  < 0.05). Moreover, there was a significant increase in the concentrations of TNF*α*, IL-1*β*, and IL-6 in the aortic tissue homogenates of HT + air group versus NT + air group (*p*  < 0.0001–*p*  < 0.05) and in HT + WPS group versus both HT + air (*p*  < 0.0001–*p*  < 0.001) and NT + WPS groups (*p*  < 0.0001) (Figure 6).

### 3.8. 8-Isoprostane, Catalase, and Total NO Levels in the Aorta Following the Exposure to WPS in NT and HT Mice

**Figure 7:**
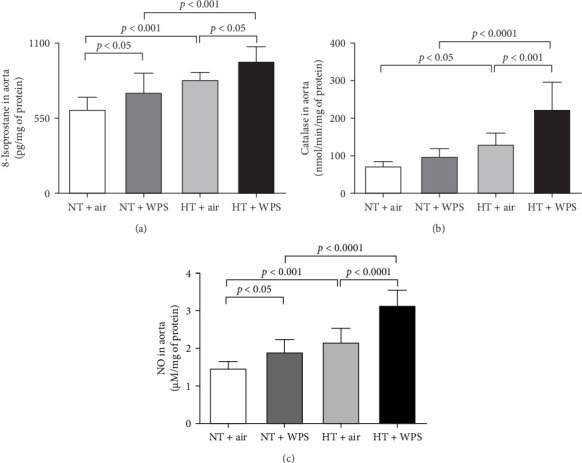
8-Isoprostane (A), catalase (B), and total nitric oxide (NO; C) in aortic tissue following exposure to either air or waterpipe smoke (WPS) in normotensive (NT) and hypertensive (HT) mice. Data are mean ± SD (*n* = 8 in each group). Statistical analysis by one-way ANOVA followed by Holm–Sidak's multiple comparisons test. ANOVA, analysis of variance.

Figure 7 shows that the levels of 8-isoprostane and total NO in aortic homogenates were elevated in NT + WPS group compared with NT + WPS group (*p*  < 0.05), and the levels of 8-isoprostane, catalase, and total NO were all augmented in HT + air group versus NT + air group (*p*  < 0.001–*p*  < 0.05). Additionally, all the measured markers of oxidative stress were significantly augmented in HT + WPS mice compared with both HT + air (*p*  < 0.0001–*p*  < 0.05) and NT + WPS (*p*  < 0.0001–*p*  < 0.001).

### 3.9. DNA Damage and Levels of Cleaved Caspase-3 and Cytochrome C in the Aorta Following the Exposure to WPS in NT and HT Mice

**Figure 8:**
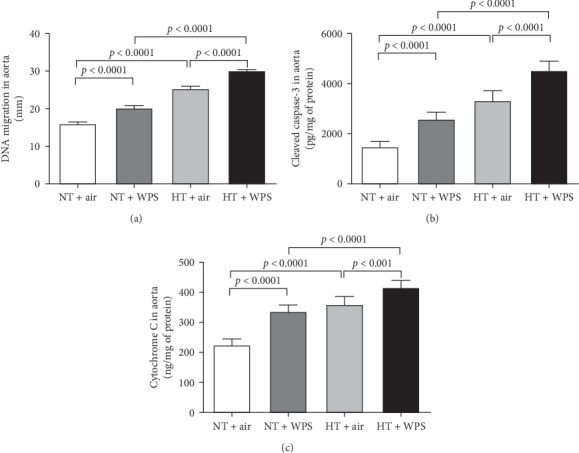
DNA damage (A) and levels of cleaved caspase-3 (B) and cytochrome (C) in aortic tissue following exposure to either air or waterpipe smoke (WPS) in normotensive (NT) and hypertensive (HT) mice. Data are mean ± SD (*n* = 5 for DNA damage assessment and *n* = 8 for cleaved caspase-3 and cytochrome C measurements). Statistical analysis by one-way ANOVA followed by Holm–Sidak's multiple comparisons test. ANOVA, analysis of variance.

The assessment of aortic DNA damage by comet assay and the markers of apoptosis-cleaved caspase-3 and cytochrome 3 revealed a significant increase in DNA migration in NT + WPS versus NT + air (*p*  < 0.0001). Consistently, the DNA migration and the levels of caspase-3 and cytochrome C were significantly increased in HT + air versus NT + air (*p*  < 0.0001) and in HT + WPS compared with both HT + air (*p*  < 0.001–*p*  < 0.0001) and NT + WPS (*p*  < 0.0001) (Figure 8).

### 3.10. Histology and Immunohistochemistry of the Aorta Following the Exposure to WPS in NT and HT Mice

**Figure 9:**
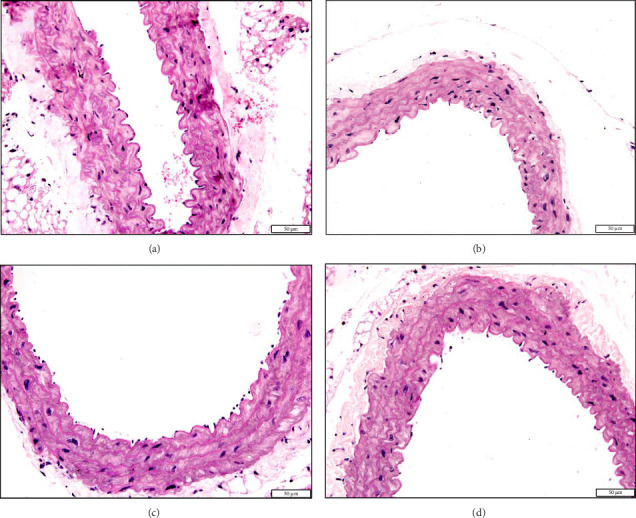
Representative light microscopy sections of aortic tissues stained with hematoxylin and eosin and obtained from normotensive (NT) and hypertensive (HT) mice after exposure to either air or waterpipe smoke (WPS). (A) Aortic section obtained from NT mice exposed to air showing normal architecture and histology. (B) Aortic section obtained from NT mice exposed to WPS showing normal architecture and histology. (C) Aortic section obtained from HT mice exposed to air showing normal architecture and histology. (D) Aortic section obtained from HT mice exposed to WPS showing normal architecture and histology.

The analysis of the aortic sections stained with H&E by light microscopy showed unremarkable aorta morphology and architecture in NT + air, NT + WPS, HT + air, and HT + WPS (Figure 9).

**Figure 10:**
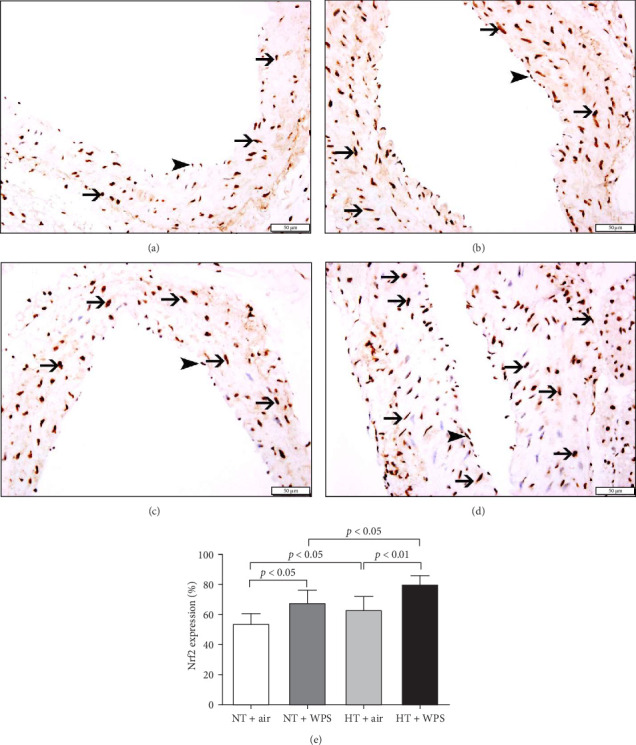
Immunohistochemical examination of the aortic tissue sections for the detection of nuclear factor erythroid-derived 2-like 2 (Nrf2) normotensive (NT) and hypertensive (HT) mice after exposure to either air or waterpipe smoke (WPS). (A) Representative section of the aorta of NT + air group showing nuclear expression of Nrf2 by smooth muscle cells (thin arrow) and endothelial cells (arrowhead). (B) Representative section of the aorta of NT + WPS group showing nuclear expression of Nrf2 by endothelial cells (arrowhead) and smooth muscle cells (thin arrow). (C) Representative section of the aorta of HT + air group showing nuclear expression of Nrf2 by endothelial cells (arrowhead) and smooth muscle cells (thin arrow). (D) Representative section of the aorta of HT + WPS group showing nuclear expression of Nrf2 by endothelial cells (arrowhead) and smooth muscle cells (thin arrow). (E) Semiquantitative assessment of the % immunohistochemical staining of the aortic tissue for Nrf2 in NT and HT mice after exposure to either air or WPS. Data are mean ± SD (*n* = 6). Scale bars in (A, B): 50 μm.

The immunohistochemistry analysis of the aortae for the detection of Nrf2 in NT and HT mice exposed to either air or WPS is shown in Figure 10. The nuclear expression of Nrf2 by endothelial lining cells and smooth muscle cells was significantly increased in NT + WPS compared with NT + air (*p*  < 0.05) and in HT + WPS group versus either HT + air (*p*  < 0.01) or NT + WPS (*p*  < 0.05) (Figure 10).

## 4. Discussion

The present work shows that WPS inhalation intensified thrombogenicity both in vivo and in vitro and vascular inflammation, oxidative stress, DNA damage, apoptosis, and Nrf2 expression in experimentally induced hypertension.

Hypertension is frequently associated with alterations in platelet activity and coagulation, leading to a prothrombotic state [26–28]. The abnormalities in platelet activation, coagulation, and fibrinolysis commonly seen in hypertension likely contribute to the increased risk of clot formation in this group of patients [28]. Studies have indicated a connection between the activation of the renin–angiotensin system and its central mediator, Ang II, with the prothrombotic condition observed in hypertension [5, 26]. This concept was confirmed in clinical practice, as prothrombotic events occur much less in hypertensive patients treated with angiotensin-converting enzyme inhibitors or Ang II receptor blockers [5, 26–28]. Furthermore, experiments involving the administration of Ang II to animals have replicated many of the platelet function irregularities and increased clotting observed in the large arteries of hypertensive patients [5, 26].

The use of tobacco products is a major cause of preventable mortality and morbidity [29]. Globally, more smokers die from cardiovascular disease than from pulmonary disease or cancer [30]. In addition to affecting various forms of cardiovascular diseases, tobacco smoking is an independent risk factor for cardiovascular disease which can distinctly induce cardiovascular adverse effects [30]. Since it is well recognized that the prevalence of tobacco smoking is elevated among hypertensive patients [14–16], we thought that it would be of interest to assess to what degree exposure to WPS aggravates the hypercoagulability state in a well-established murine model of hypertension. Such interaction has not been investigated before.

We, and others, have previously reported that exposure to WPS induces prothrombotic events in mice [11, 19, 31]. However, it is not known whether these effects are aggravated in experimentally induced hypertension. In this work, we have studied the impact of WPS on coagulation events in HT and NT by evaluating a set of relevant indices, that is, plasma concentrations of tissue factor, vWF, fibrinogen, and PAI-1 and platelet aggregation both in vivo and in vitro and PT and aPTT. Our data show that the concentrations of various plasma markers of hemostasis were substantially increased in HT mice exposed to WPS compared with both HT mice exposed to air and NT mice exposed to WPS. The latter markers included tissue factor which is expressed in subendothelial cells upon injury, vWF that mirrors endothelial cell release and vascular reactivity, fibrinogen which is an acute-phase protein that increases blood viscosity and coagulation and PAI-1 which is an effective endogenous inhibitor of fibrinolysis [32–35]. Moreover, we found that the thrombotic occlusion time in pial arterioles and venules in vivo and platelet aggregation in whole blood in vitro was significantly potentiated in hypertensive mice exposed to WPS compared with the controls. Likewise, PT and aPTT were significantly reduced in HT + WPS compared with both NT + WPS and HT + air indicating hypercoagulability. Such actions were not reported before. It has been previously demonstrated that pulmonary exposure to diesel exhaust particles exacerbates thrombotic events in mice with hypertension [8]. Also, it is well-known that individuals with hypertension, diabetes, or dyslipidemia are vulnerable to developing thromboembolic complications [36]. Moreover, it has been shown that smoking independently increases the risk of thromboembolic events, and given its prevalence in hypertensive patients, it contributes as an additive causal factor to thromboembolic events [37].

Endothelial dysfunction manifests as a shift in endothelial activities, leading to diminished vasodilation, elevated platelet adhesion and activation, and a proinflammatory and prothrombotic state. This dysfunction is closely linked to various cardiovascular risk factors, such as hypertension, which drives inflammation within the vascular wall, promoting the expression of cell adhesion molecules and increasing the risk of thrombosis [38]. It has been recently reported that the exposure to WPS increased the expression of cell adhesion molecules including intercellular adhesion molecule-1, vascular cell adhesion molecule-1, E-selectin, and P-selectin in the aorta of healthy mice [22]. In the present study, we confirmed the increase in the expression of P- and E-selectins and ICAM-1 and VCAM-1 in NT mice exposed to WPS compared to those exposed to air and have shown, for the first time, that this effect is potentiated in HT mice exposed to WPS compared with both NT + WPS and HT + air. Along with the overexpression of cell adhesion molecules, our data demonstrate the occurrence of inflammation characterized by the increase of concentrations of proinflammatory cytokines (TNF*α*, IL-1*β*, and IL-6) and oxidative stress markers (the marker of lipid peroxidation 8-isoprostane, the antioxidant catalase, and the free radical scavenger NO) in the aortas of HT mice exposed to WPS compared with the controls. The latter indicates the occurrence of vascular injury following the exposure to WPS which is aggravated in HT mice. Both clinical and experimental studies have demonstrated that WPS inhalation induces vascular damage [9, 22, 39, 40].

It is well-recognized that inflammation and oxidative stress potentiate each other and are both important sources of DNA injury, as augmented levels of reactive oxygen species have been shown to induce oxidative DNA damage [41–46]. Moreover, it is well-established that if the DNA injury is not repaired, cells could undergo programmed cell death or apoptosis [41, 42]. Large body of clinical evidence has reported that oxidative stress is involved in the pathogenesis of hypertension and that DNA damage induced by reactive oxygen species happens more frequently in hypertensive people than in NT ones [47, 48]. In line with clinical studies, our data confirm that DNA damage and markers of apoptosis including cleaved caspase-3 and cytochrome C are increased in HT mice exposed to air compared with NT mice exposed to air. More importantly, here, we have shown that inhalation of WPS in HT mice consistently exaggerated DNA damage and apoptosis when compared with HT mice exposed to air and NT ones exposed to WPS.

Nrf2 is a vital transcription factor that plays a significant role in activating antioxidant enzymes following oxidative stress occurrences. Upon encountering oxidative stress, Nrf2 is released from the regulatory Keap1–Nrf2 complex, relocating from the cytoplasm to the nucleus, where it binds to the antioxidant response element, a regulatory enhancer region in gene promoters. This binding initiates the production of antioxidant enzymes, offering protection against cell damage induced by oxidative stress [49, 50]. The regulation of cellular antioxidant and anti-inflammatory machinery by Nrf2 is essential for counteracting oxidative stress. In this context, it has been shown that Nrf2 disruption in mice alleviates or annuls the initiation of genes encoding antioxidant molecules following oxidative stress [51]. Furthermore, it has been reported that inhalation of WPS induces an overexpression of Nrf2 in the heart, and the concomitant administration of a natural antioxidant gum Acacia further elevates the expression of Nrf2 [25]. However, the expression of Nrf2 in aortic tissue of HT exposed to WPS has not been reported before. Our data show that in comparison with both NT mice exposed to WPS and HT animals exposed to air, the aortic specimens obtained from mice with HT and exposed to WPS revealed a notable rise in the nuclear expression of Nrf2 by endothelial lining cells and smooth muscle cells in the aortic sections. The latter findings along with the increase in the expression of the antioxidant catalase could be explained by a possible compensatory effect aiming at counterbalancing the exaggerated level of oxidative stress seen HT + WPS group (increase of 8-isoprostane and NO) [24, 52]. Our data show no change in aortic morphology by H&E staining between the studied groups. This observation is in agreement with previous studies reporting that exposure to WPS or nanoparticles induces biochemical, physiological, and immunohistological changes in various organs including the heart but without altering the organ morphology [18, 19, 25]. The latter suggests that the aforementioned changes precede the morphological alteration which could be seen in mice after long-term exposure to WPS.

The current study has limitations regarding the specific mechanisms of WPS injury and the hierarchical pathogenetic processes involving coagulation, cell injury, and oxidative stress. Future research is needed to comprehensively investigate these processes and their role in the pathogenesis of WPS-related thrombotic and vascular toxicity in hypertension.

## 5. Conclusions

In conclusion, our data demonstrate that WPS exposure intensified thrombogenicity and vascular inflammation, oxidative stress, DNA damage, apoptosis, and Nrf2 expression in experimentally induced hypertension. Our data suggest that vascular toxicity of WPS may be exaggerated in individuals with hypertension. This possibility, however, needs to be tested in well-controlled human studies.

## Figures and Tables

**Table 1:** Systolic blood pressure (SBP) in normotensive (NT) and hypertensive (HT) mice after inhalation of either air or waterpipe smoke (WPS).

Parameter assessed/Groups	NT + air	NT + WPS	HT + air	HT + WPS
Systolic blood pressure	87.1 ± 2.8	124.6 ± 1.5*⁣*^*∗∗∗∗*^	134.9 ± 4.0*⁣*^*∗∗∗∗*^	158.6 ± 3.6^ΔΔΔΔ,^⁣^*∗∗∗∗∗*^

*Note:* Data are mean ± SD (*n* = 8 in each group). Statistical analysis by one-way ANOVA followed by Holm–Sidak's multiple comparisons test.

Abbreviations: NT, normotensive; WPS, waterpipe smoke.

*⁣* ^*∗∗∗∗*^ *p*  < 0.0001 compared with NT + air group.

^ΔΔΔΔ^ *p*  < 0.0001 compared with HT + air.

⁣^*∗∗∗∗∗*^*p*  < 0.0001 compared with NT + WPS.

## Data Availability

The data that support the findings of this study are available from the corresponding author upon reasonable request.
